# Supplementary material for: Structural basis of the dynamic human CEACAM1 monomer-dimer equilibrium
Source: Commun Biol. 2021 Mar 19;4:360. doi: 10.1038/s42003-021-01871-2 (PMC7979749; doi:10.1038/s42003-021-01871-2)
Supplement: Supplementary file 2 — Supplementary Information [file 42003_2021_1871_MOESM2_ESM.pdf]

Supplementary Information

Structural basis of the dynamic human CEACAM1 monomer-dimer equilibrium

Amit K. Gandhi[1,11*], Zhen-Yu J. Sun[2,11], Walter M. Kim[1,11], Yu-Hwa Huang[1,11], Yasuyuki Kondo[1,3], Daniel A. Bonsor[4], Eric J. Sundberg[4,5,6,7], Gerhard Wagner[8], Vijay K. Kuchroo[9], Gregory A. Petsko[10], Richard S. Blumberg[1*]

[1] Division of Gastroenterology, Department of Medicine, Brigham and Women's Hospital, Harvard Medical School, 75 Francis Street, Boston, MA 02115, USA.
[2] Department of Cancer Biology, Dana-Farber Cancer Institute, Boston, MA 02215, USA.
[3] Current address: Division of Gastroenterology, Department of Internal Medicine, Graduate School of Medicine, Kobe University, Kobe, 650-0017, Japan.
[4] Institute of Human Virology, University of Maryland School of Medicine, University of Maryland, 725 W Lombard St, Baltimore, MD 21201, USA.
[5] Department of Medicine, University of Maryland School of Medicine, University of Maryland, Baltimore, MD 21201,USA.
[6] Department of Microbiology and Immunology, University of Maryland School of Medicine, University of Maryland, Baltimore, MD 21201, USA.
[7] Current address: Department of Biochemistry, Emory University School of Medicine, Atlanta, GA 30322, USA.
[8] Department of Biological Chemistry and Molecular Pharmacology, Harvard Medical School, 240 Longwood Avenue, Boston, MA, 02115, USA.
[9] Evergrande Center for Immunologic Diseases and Ann Romney Center for Neurologic Diseases, Harvard Medical School and Brigham and Women's Hospital, 77 Avenue Louis Pasteur, Boston, MA 02115, USA.
[10] Ann Romney Center for Neurologic Diseases, Department of Neurology, Brigham and Women's Hospital, Harvard Medical School, Boston, MA 02115, USA.
[11]These authors contributed equally to this work.
* For correspondence: rblumberg@bwh.harvard.edu, agandhi2@bwh.harvard.edu

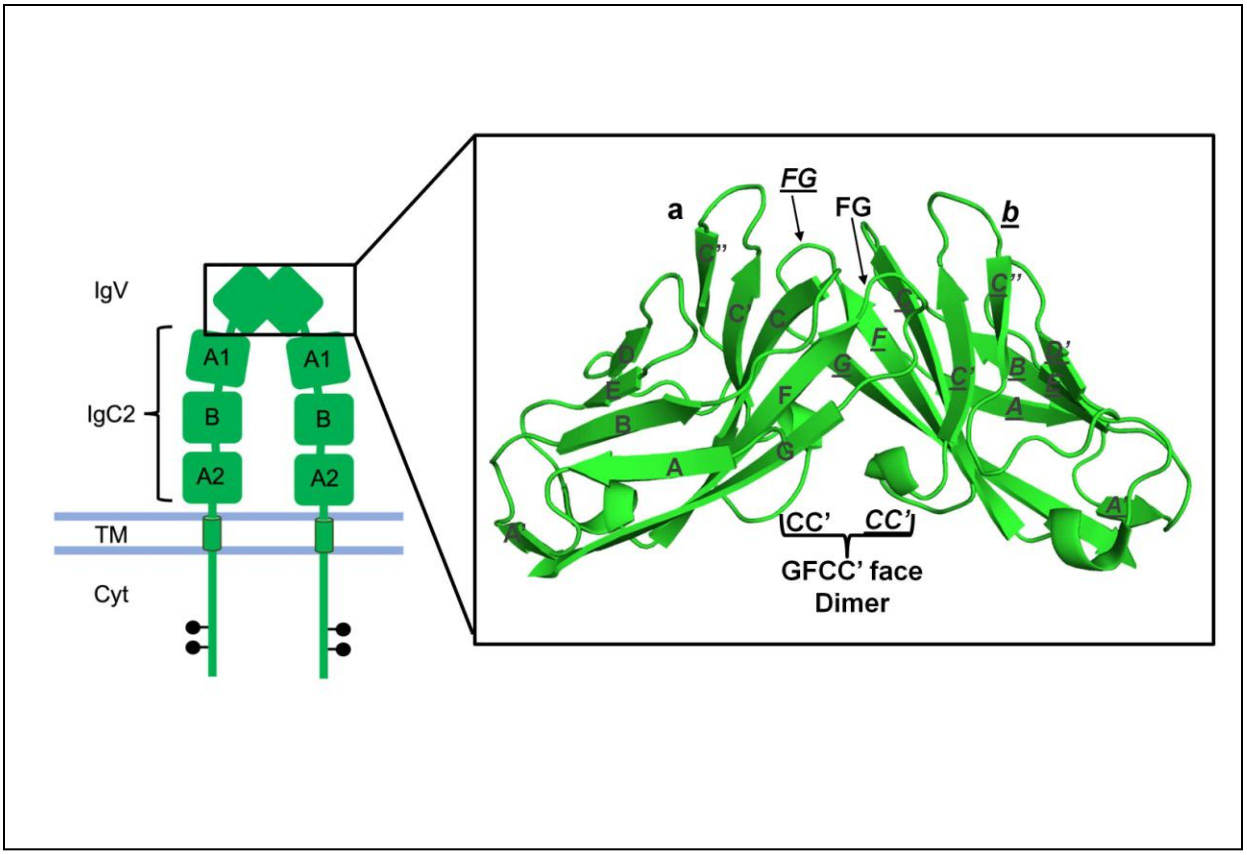

Supplementary Fig. 1. **Domain structure of the hCEACAM1**. The human CEACAM1 domain structure (green) contains an N-terminal IgV domain followed by three IgC2 domains (A1, B, A2) coupled to a transmembrane sequence and a long tail (shown here) with two immune receptor tyrosine-based inhibitory motifs (stick and ball symbol) or short cytoplasmic tail. The insert shows a ribbon diagram of an GFCC'-mediated IgV domain dimer with each domain containing two anti-parallel β-sheet sandwich faces formed by front AGFCC′C″ and back BED faces, respectively. The IgV domain of human CEACAM1 mediates GFCC' face homodimer formation and interactions with various ligands such as human TIM-3 and HopQ through the GFCC' face wherein residues of CC' and FG loop are involved in the interactions. The two monomers that form the homodimer are indicated a and *b* (italic and underlined), respectively. The β strands and loops are labeled with uppercase letters and underlined italic for monomers a and *b, respectively.*

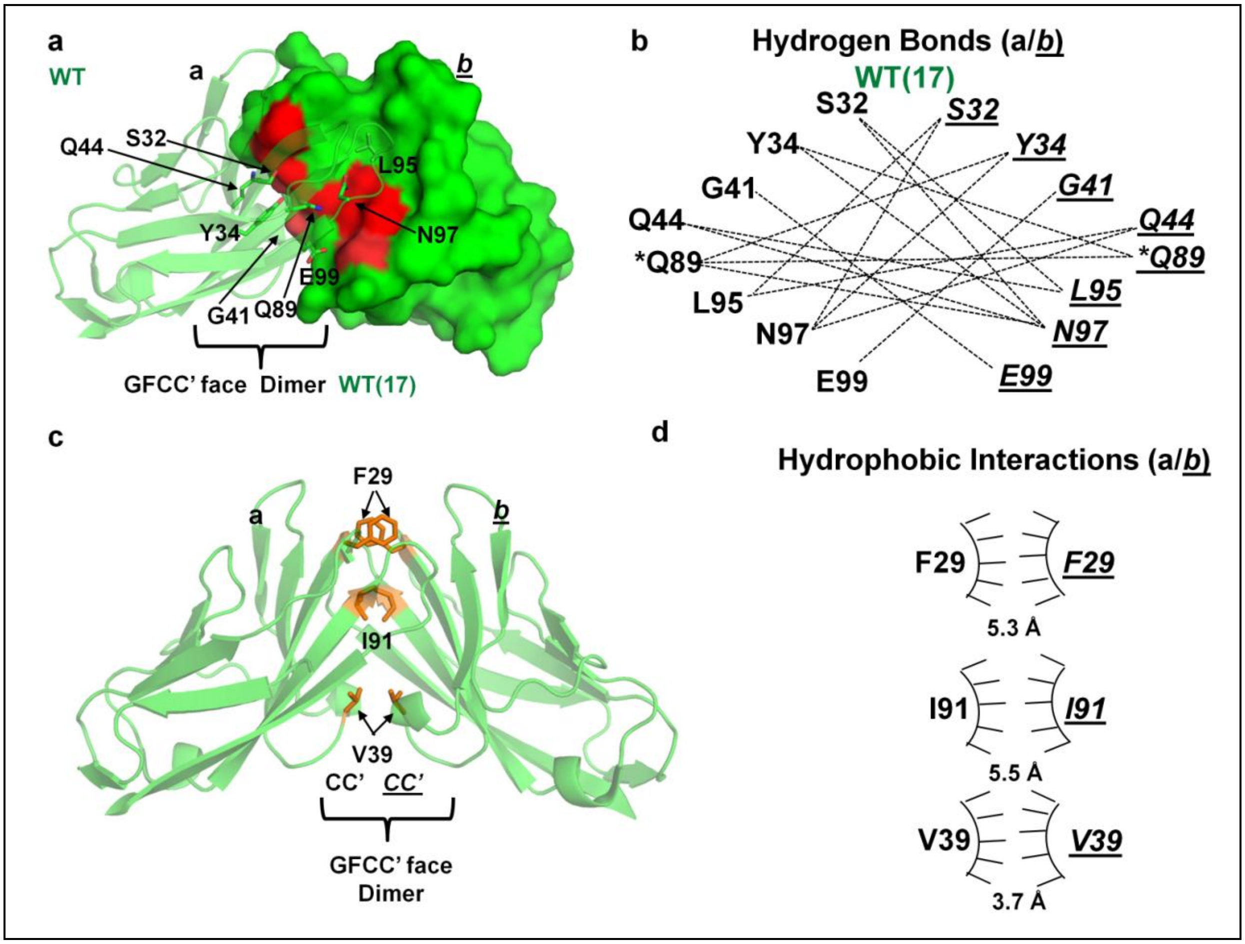

Supplementary Fig. 2. Crystal structure of the hCEACAM1 IgV WT homodimer (PDB code 4QXW, resolution (2.04 Å) with quantification of hydrogen bonds and hydrophobic interactions at the GFCC' face. a Ribbon (molecule a) and surface diagram (molecule *b*) of the hCEACAM1 IgV crystal structure in green. The residues S32, Y34, G41, Q44, Q89, L95, N97, and E99 mediate seventeen (17) hydrogen bonded interactions to form the GFCC' face homodimer and are shown by stick and surface (bright and light red) representation for molecule (a) and (*b*), respectively. **b** Hydrogen bonded interactions of GFCC' face residues. The molecule (*b*) residues are labeled in italics and underlined. The hydrogen bonded interactions (17) across the GFCC' face by residues described above are shown by dashed lines. The asterisk (*)

indicates two hydrogen bonds (shown by single dashed line) mediated by Q89 residues of molecules (a) and (*b*) with each other via OE1 and NE2 atoms. **c** The ribbon diagram of the hCEACAM1 (green) with residues F29, I91, and V39 of molecule (a) and (*b*) that mediate strong hydrophobic interactions in the formation of GFCC' face dimer are shown by orange stick representation. The CC' loops are labeled. **d** The hydrophobic interactions of GFCC' face F29, I91, and V39 residues are shown by arc/point representations. The molecule (a) and (*b*) residues are labeled with hydrophobic interactions as measured by distance between β carbons of labeled residues of molecules (a) and (*b*).

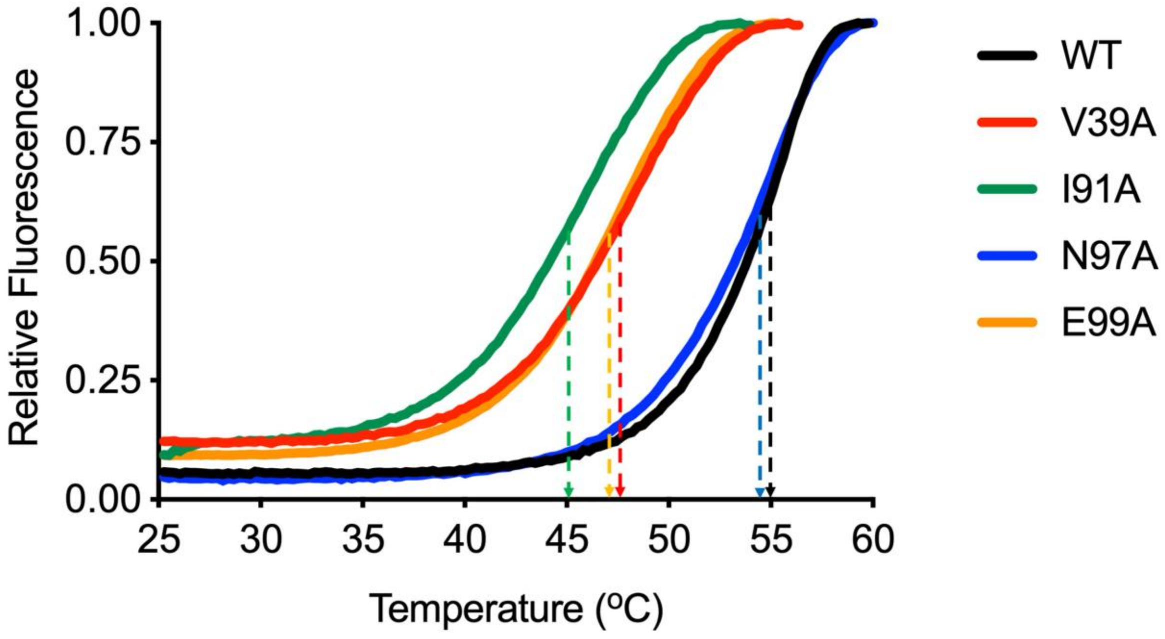

Supplementary Fig. 3. Differential scanning fluorimetry of wildtype (WT) and mutant hCEACAM1 IgV. Relative absorbance emission with corresponding temperature is plotted for WT (black) hCEACAM1 IgV and V39A (red), I91A (green), N97A (blue), E99A (orange) mutant hCEACAM1 IgV protein samples. Melting temperature (T_M) calculated by first derivative analysis is designated for each curve by colored arrow. Each curve is representative of triplicate samples.

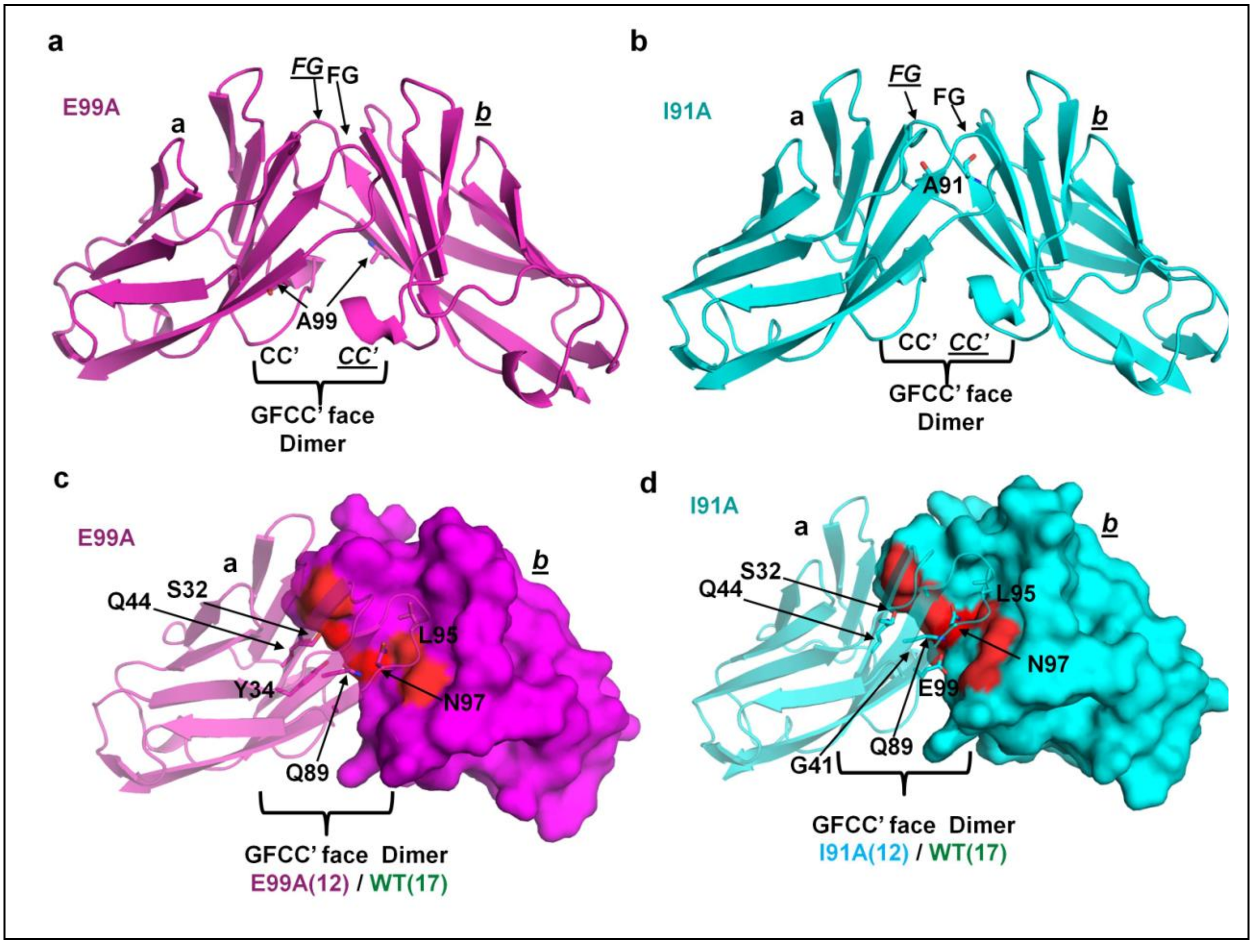

Supplementary Fig. 4. Crystal structures of the E99A and I91A IgV mutants of hCEACAM1. a Ribbon diagram (magenta) of the molecule (a) and molecule (*b*) as observed in the unit cell of the E99A crystal structure. The A99 residues of molecule (a) and molecule (*b*) are shown by stick representation. The FG and CC' loops that mediates formation of GFCC' face dimer are labeled in bold for the molecule (a) and labeled in italics and underlined for the molecule (*b*). The carbon atoms in magenta, carbonyl oxygen in red and nitrogen in blue, are colored, respectively**. b** Ribbon diagram (cyan) of the molecule (a) and molecule (*b*) as observed in the unit cell of the I91A crystal structure. The A91 residues of molecule (a) and molecule (*b*) are shown by stick representation. The FG and CC' loops are labeled as described above. The carbon atoms in cyan, carbonyl oxygen in red and nitrogen in blue, are colored, respectively. **c**

The ribbon and surface diagram of the molecule (a) and (*b*) in magenta as observed in the E99A crystal structure. The S32, Y34, Q44, Q89, L95, and N97 residues that mediate hydrogen bonded interactions in the formation of GFCC' face dimer of E99A crystal structure are shown by stick and surface bright and light red representations. The lesser quantity of hydrogen bonds at GFCC' interface were observed in the E99A mutant structure, 12 (magenta) vs 17 (green) for E99A and WT, respectively. The carbon atoms in magenta, carbonyl oxygen in red and nitrogen in blue, are colored, respectively**. d** The ribbon and surface diagram of the molecule (a) and (*b*) in cyan as observed in the I91A crystal structure. The S32, G41, Q44, Q89, L95, N97, and E99 residues that mediates hydrogen bonded interactions in the formation of GFCC' face dimer of I91 crystal structure are shown by stick and surface bright and light red representations for molecule (a) and (*b*), respectively. The lesser quantity of hydrogen bonds at GFCC' interface were observed in the I91A mutant structure, 12 (cyan) vs 17 (green) for I91A and WT, respectively. The carbon atoms in cyan, carbonyl oxygen in red and nitrogen in blue, are colored, respectively.

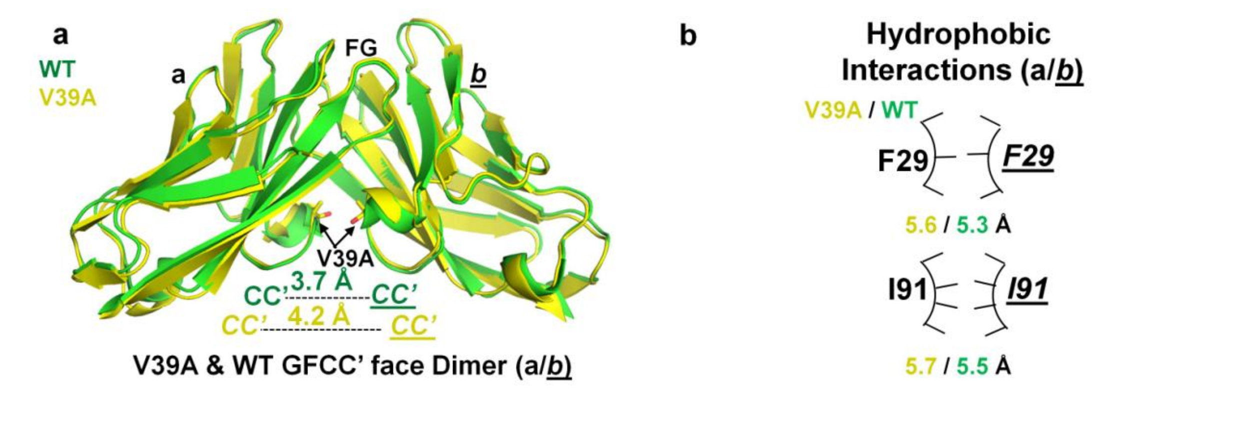

Supplementary Fig. 5. V39A mutant GFCC' face dimer. **a** The V39A mutant dimer formed by molecules (a) and (*b*) mimic hCEACAM1 WT GFCC' face dimer (PDB code 4QXW). The ribbon diagram of the V39A mutant (yellow) and WT (green) IgV molecules (a) and (*b*) superimposed on each other and GFCC'C' face dimer is shown. The V39A mutation is shown by stick representation and CC' loops are labeled with distances across CC' loops for V39A mutant (4.2 Å) and WT (3.7 Å) in yellow and green, respectively. The V39A mutant carbon atoms in yellow, carbonyl oxygen in red and nitrogen in blue, are colored, respectively. The CC' loop distances are measured by the distances between β carbons of A39 in V39A mutant or distances between β carbons of V39 in WT, respectively. **b** The hydrophobic interactions of GFCC' face F29, and I91 residues are shown by arc/point representations. The molecule (*b*) residues are in italics and underlined, respectively. The hydrophobic interactions as measured by distance between β carbons of labeled residues of molecules (a) and (*b*) are shown in yellow and green, for V39A mutant GFCC' face dimer and WT, respectively.

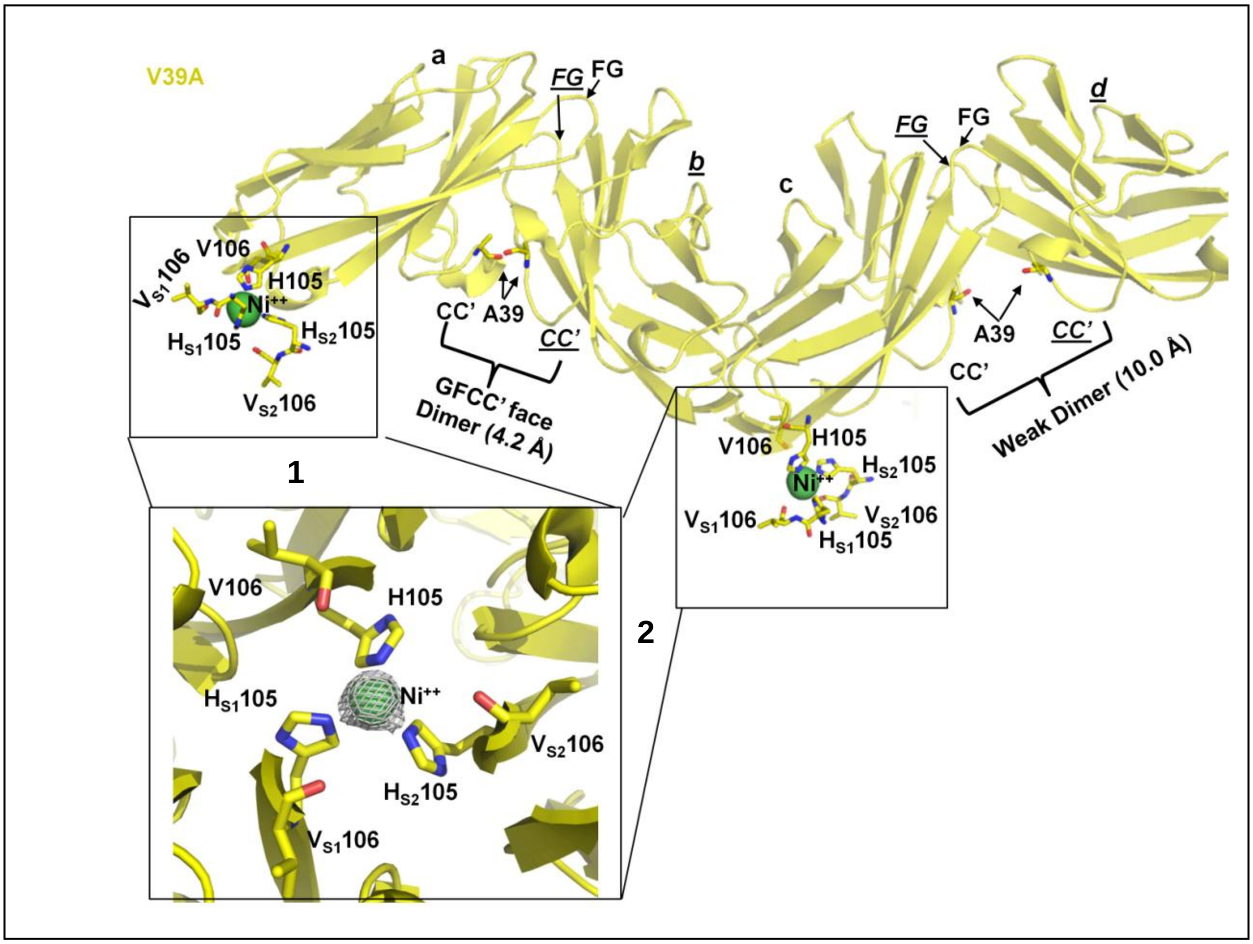

Supplementary Fig. 6. **The V39A mutant crystal structure and binding with Nickel (Ni $^{++)}$.** Overall ribbon diagram of the four molecules (a, *b*, c, *d*) in yellow, whereas A39 residue of each molecule is shown by stick representation and binding of Ni^{++} with molecule (a) or (c) and their symmetry mate residues H105 and V106. The carbon atoms in cyan, carbonyl oxygen in red and nitrogen in blue, are colored, respectively. Molecules (a) and (*b*) form a GFCC'C' face dimer that mimics a WT dimer with CC' loop distances as described supplemental Fig. 5A of 4.2 Å. In contrast, molecules (c) and (*d*) make a weak V39A dimer where the CC' loops are apart with a distance of 10.0 Å and exhibit large conformational differences compared to a WT GFCC' face dimer. The inset 1 or 2 show interactions of six V39A mutant residues H105 and V106 from molecule (a) and its two symmetry mates 0100-100 (S1) and 02000000 (S2) with bound Ni^{++} (green

sphere) as observed in the V39A crystal structure. The electron density for bound Ni^{++} is shown by 2Fo-Fc map at 4.0 σ level. Residues H105, V106 from molecule (a), $H_{S1}105$, $V_{S1}106$, from symmetry molecule a_0100-100, and $H_{S2}105$, $V_{S2}106$ from symmetry molecule a _02000000, are highlighted in stick representation. These residues make interactions with Ni^{++}, whereas nitrogen of the three histidine rings of residues H105 (2.3 Å) and three carbonyl oxygen of residues V106 (4.5 Å) participate in hexa-coordinated interactions with Ni^{++}. Similar interactions were also observed with molecule (c) and its symmetry mates in binding with a second Ni^{++} as observed in the crystal structure (2).

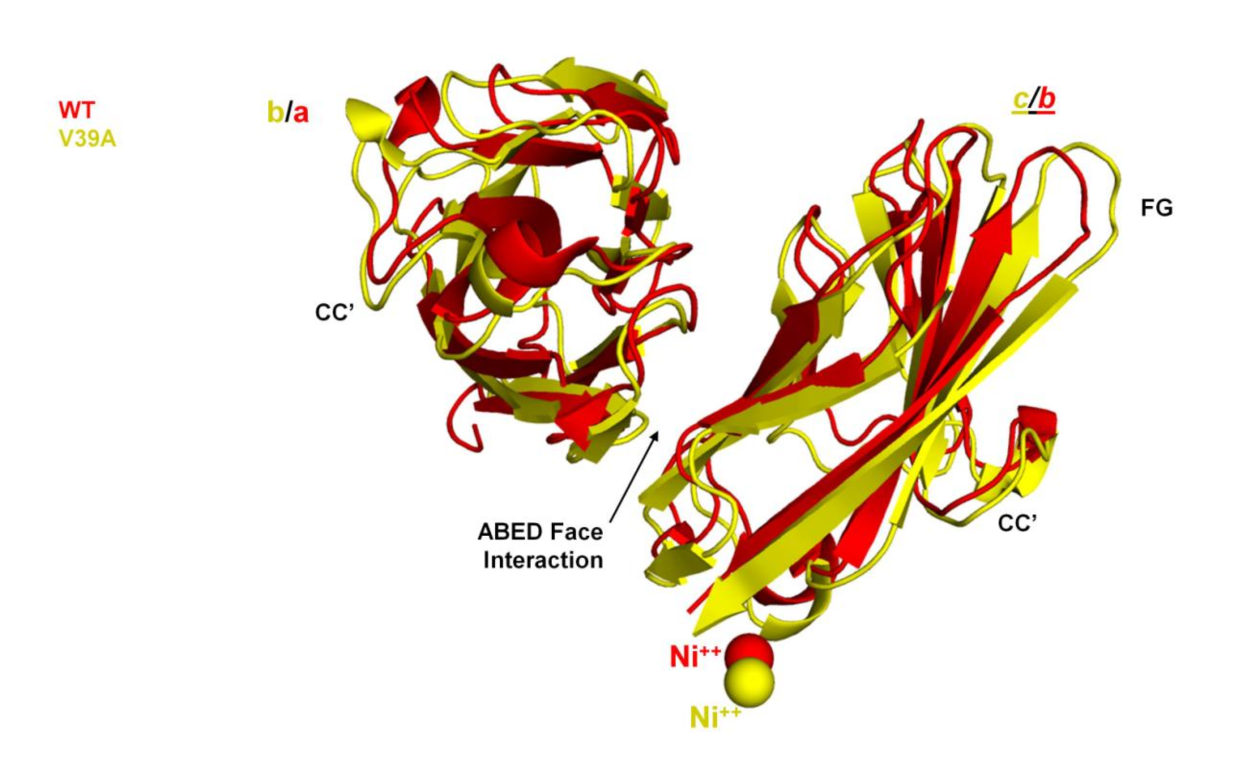

Supplementary Fig. 7. **The overall similarity of hCEACAM1 WT structure with an ABED face dimer (PDB code 2GK2) and interface formed by molecules (b) and (c) in the V39A mutant structure.** The ribbon diagram of the structural superimposition of the hCEACAM1 WT (PDB code 2GK2) structure (molecules a and *b*, colored red) and V39A mutant structure (molecules b and c, colored yellow) with with C-alpha root mean square deviation (RMSD) of 2.7 Å (over 1647 atoms). The superimposition revealed an overall similar minor ABED face contacts through ABED face residues including Y68, N70, and S72 (depicted by arrow). In addition, a similar mode of Ni^{++}, binding was observed involving residues His105 in hCEACAM1 WT (PDB code 2GK2) structure (molecules *b*) and V39A mutant structure (molecules c). The bound Ni^{++} is shown by sphere and colored red and yellow for hCEACAM1 WT (PDB code 2GK2) and V39A mutant, respectively. The CC' and FG loops are labeled.

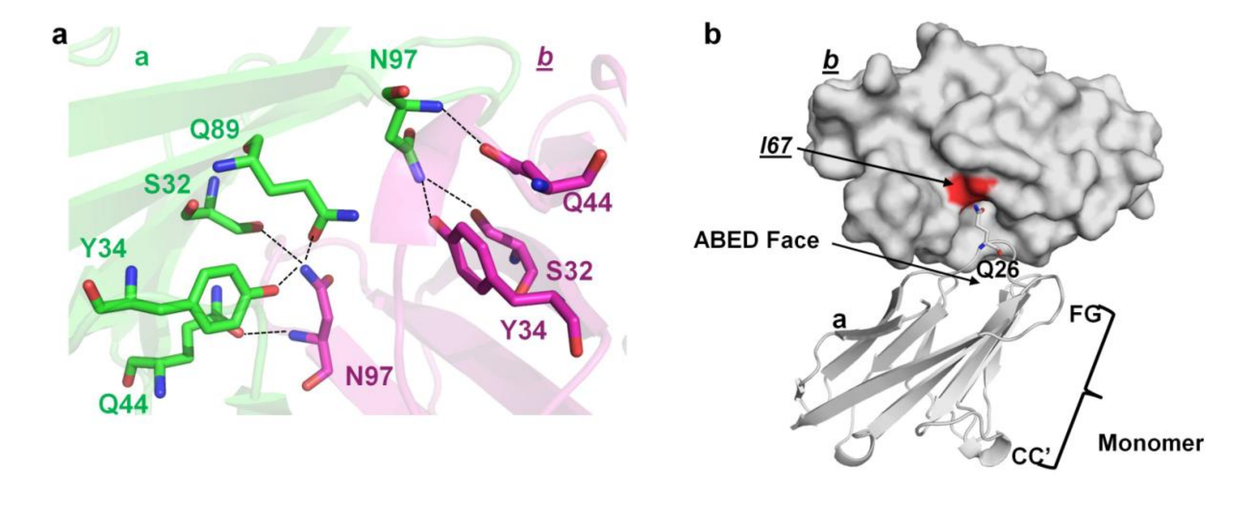

Supplementary Fig. 8. **N97-mediated asymmetrical hydrogen bonded interactions in the wild type and absence in monomeric N97A mutant crystal structure. a** Seven asymmetrical hydrogen bonded interactions (dashed lines) mediated by N97A residues of molecule a in green and molecule (*b*) in magenta as observed in the hCEACAM1 WT homodimer structure (PDB code 4QXW). The N97 residue from molecule (a) makes three hydrogen bonded interactions with residues S32, Y34, and Q44 of molecule (*b*) and N97 residue from molecule (*b*) makes four hydrogen bonded interactions with residues S32, Y34, Q44, and Q89 of molecule (a). The residues are shown by stick representation and hydrogen bonds are shown by dashed lines. The molecule (a) carbon atoms in green, carbonyl oxygen in red and nitrogen in blue, are colored, respectively. The molecule (b) carbon atoms in magenta, carbonyl oxygen in red and nitrogen in blue, are colored, respectively. **b** The ribbon and surface diagram (silver white) of N97A monomeric molecules (a) and (*b*), respectively, as observed in the unit cell of N97A crystal structure. The residues Q26 (stick representation) of molecule (a) and I67 (surface colored red) of molecule (*b*) make two hydrogen bonded interactions and form a minor point of contact between two molecules at ABED face (shown by an arrow). The molecule (a) residue Q26 is shown by stick representation, whereas carbon atoms in silver white, carbonyl oxygen in red and nitrogen in blue, are colored, respectively. The CC' and FG loops of molecule (a) is labeled and absence of GFCC' face interaction confirm the monomeric nature of N97A mutant.

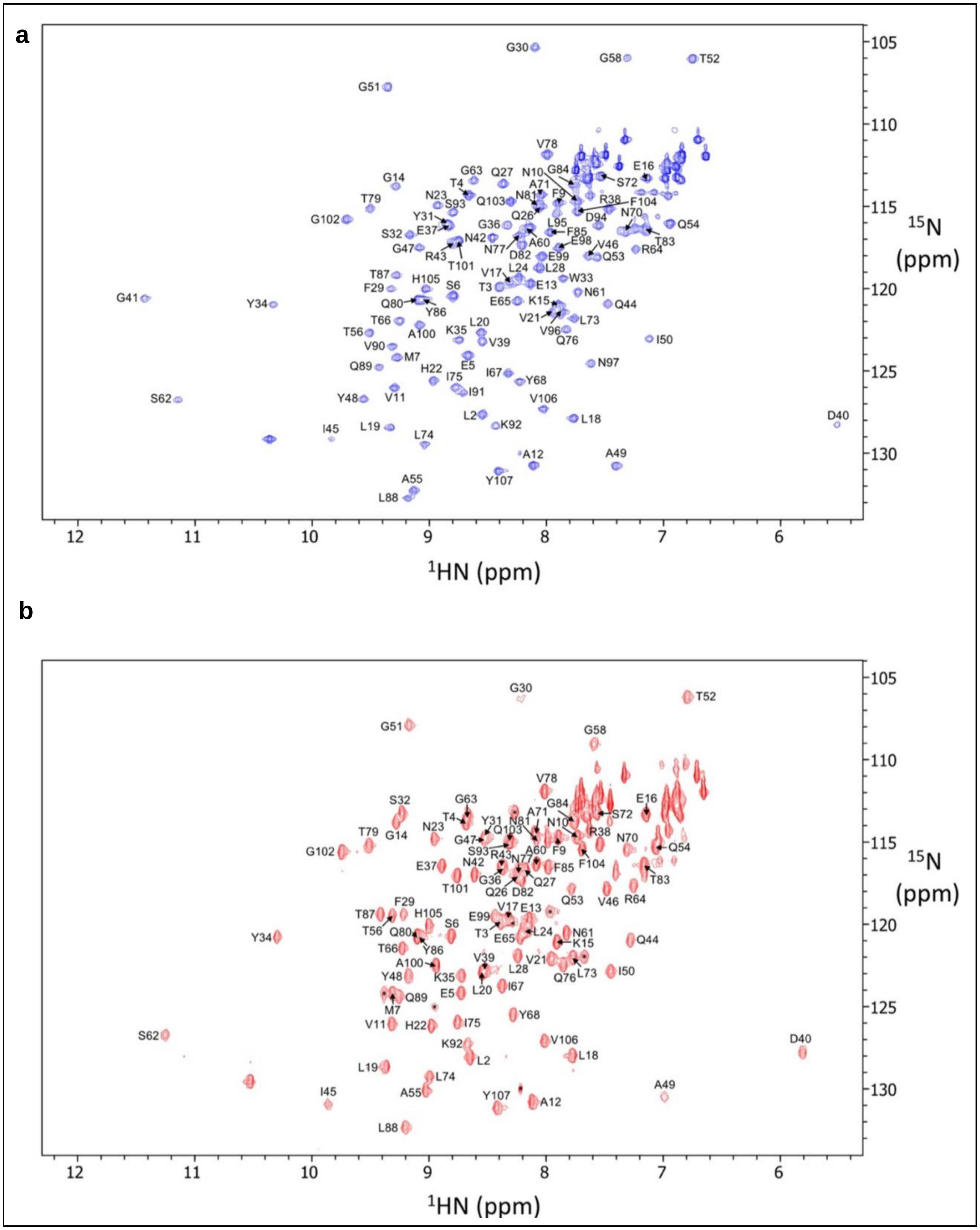

Supplementary Fig. 9. **^{15}N HSQC spectra of WT and N97A IgV mutant proteins.**

a Assigned (backbone amide) ^{15}N-HSQC spectrum of hCEACAM1 WT protein (blue). **b** Assigned (backbone amide) ^{15}N-HSQC spectrum of N97A mutant (red) protein.

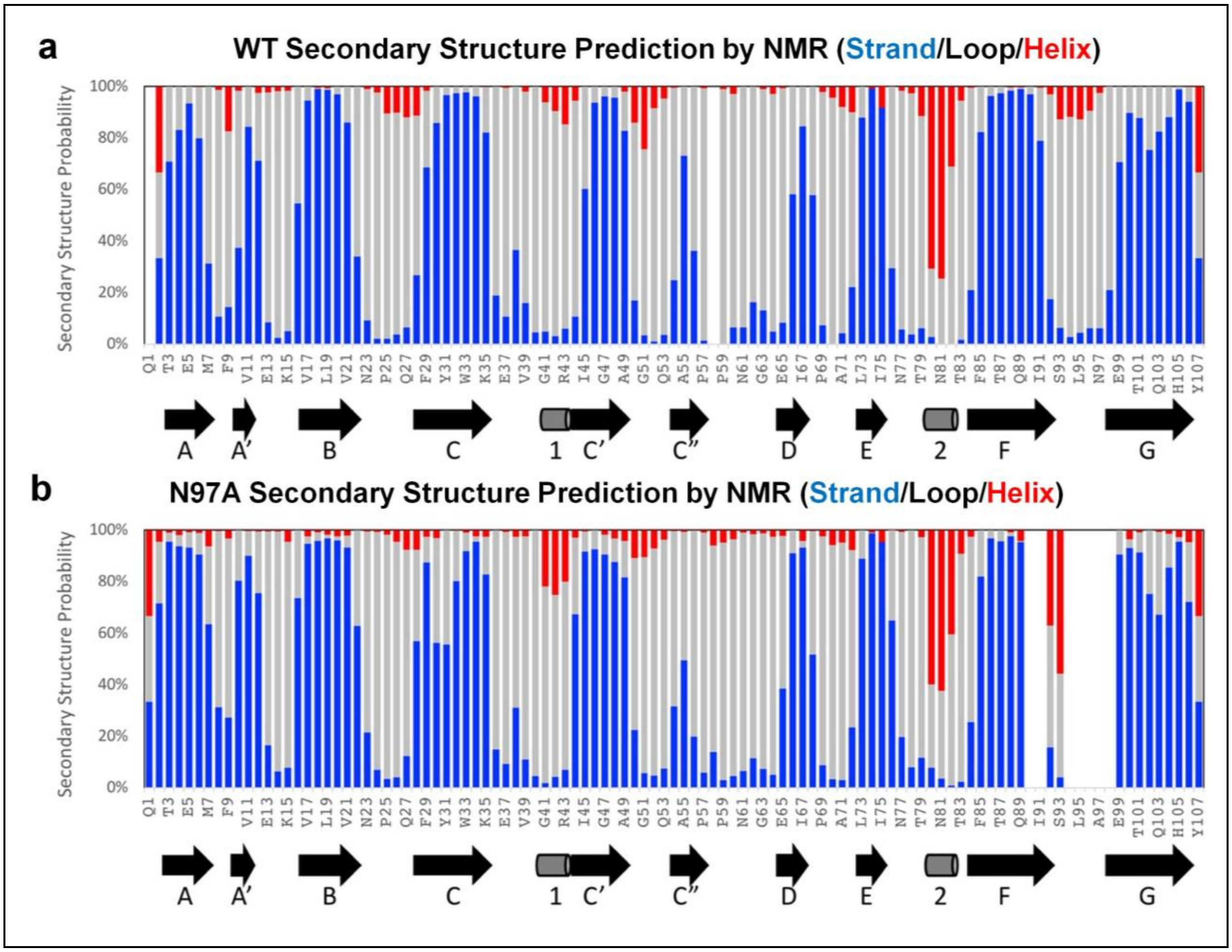

Supplementary Fig. 10. **hCEACAM1 WT IgV and N97A NMR secondary structures.** **a** Secondary structure probabilities of hCEACAM1 WT IgV predicted from NMR chemical shift values, in comparison with the secondary structures from the crystal structure (PDB code 4QXW) depicted below. **b** Secondary structure probabilities of N97A mutant hCEACAM1 IgV predicted from NMR chemical shift values, in comparison with the secondary structure from the X-ray structure (PDB code 6XO1) depicted below. The probabilities of predicted beta-strand, loop, and alpha-helix are colored in blue, grey, and red respectively; the black ribbons and grey cylinders represent beta strands and alpha-helices resepectively.

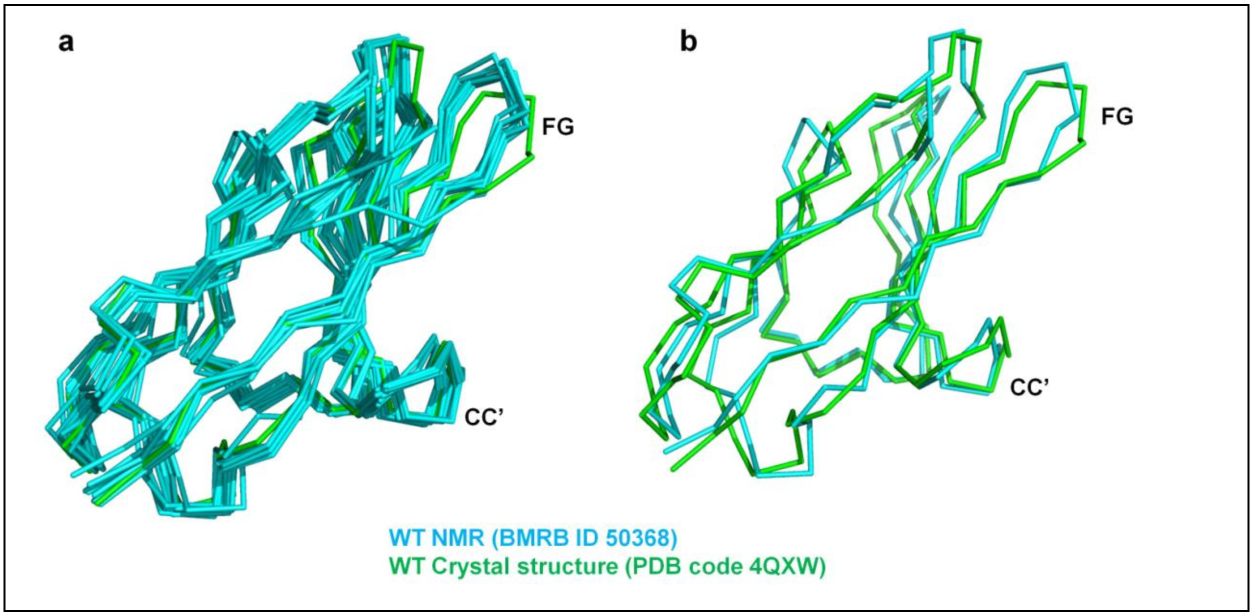

Supplementary Fig. 11. **WT NMR secondary structure prediction and comparison.** Similarity and superimposition of human CEACAM1 WT predicted NMR structure (cyan, BMRB ID 50368) and crystal structure (green, PDB code 4QXW). **a** The top ten lowest energy NMR predicted structures of hCEACAM1 WT (cyan) superimposition with the WT crystal structure (green) with a RMSD of 0.7 Å (over 664 atoms). All the ten NMR states are shown. The CC' and FG loops are labeled. **b** The superimposition of one of the NMR predicted structure of hCEACAM1 WT (cyan) superimposition with the WT crystal structure (green). Only one state is shown.

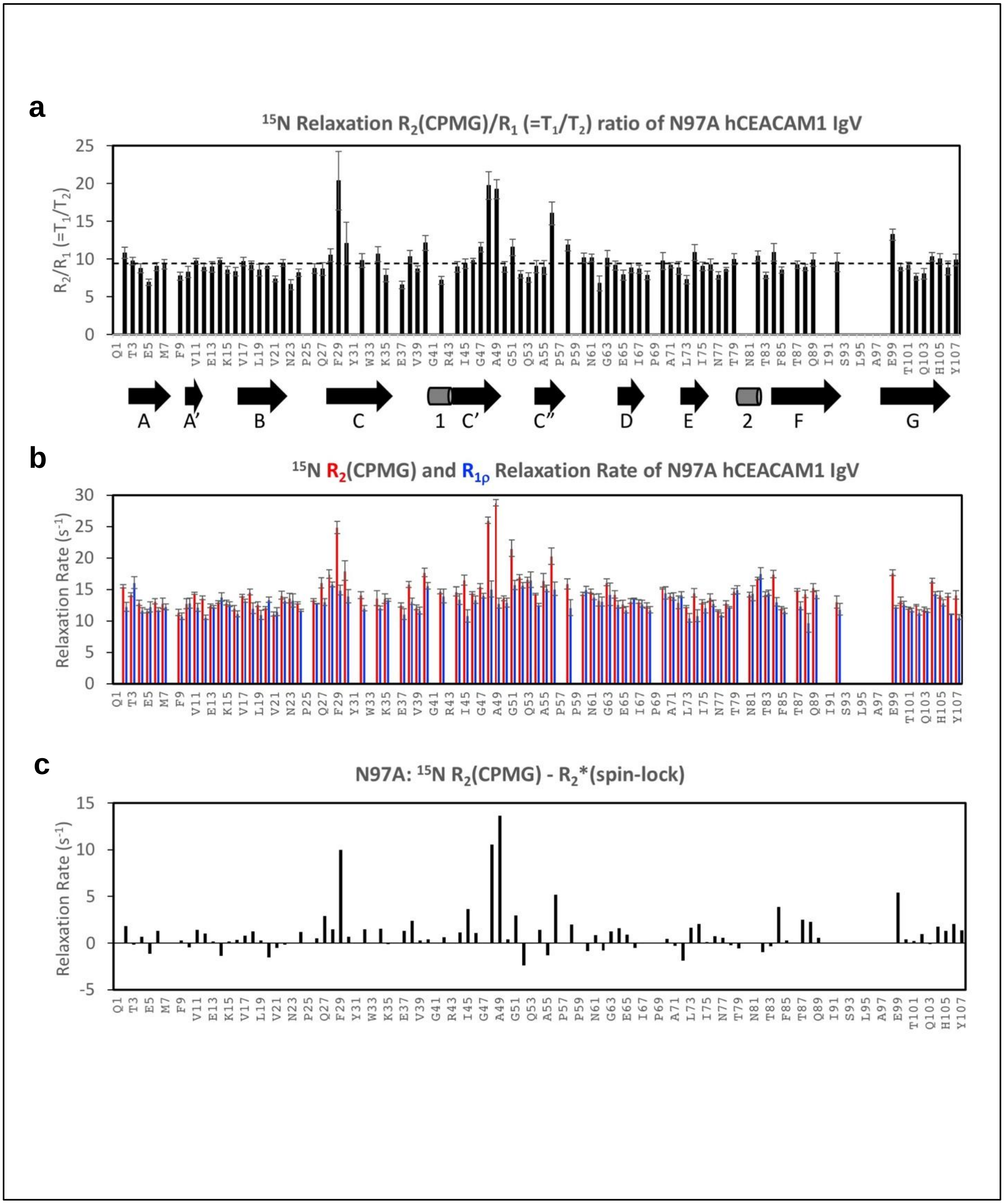

Supplementary Fig. 12 NMR relaxation rates. a The ratio of NMR relaxation rates R_2/R_1 (same as the ratio of relaxation times T_1/T_2) of N97A mutant hCEACAM1 IgV showing remarkable dynamics of several residues at the GFCC’ face. T_2 was measured

by a CPMG type experiment (with τ = 625ms). **b** The comparison between R_2 (red) and $R_{1\rho}$ (blue) NMR transverse relaxation rates of N97A mutant hCEACAM1 IgV showing chemical exchange effects that are better suppressed in the $T_{1\rho}$ experiment (with a spin-locking field strength of 1.75kHz) than in the T_2 CPMG experiment (with a CPMG τ = 625ms). **c** The difference between transverse relaxation rates R_2(CPMG) and R_2*(spin-lock) (derived from $R_{1\rho}$ according to $R_{1\rho} = R_1\cos^2\theta + R_2^*\sin^2\theta$, where θ is the off-resonance tilt angle of the effective spin-locking field in the rotating frame) of N97A mutant hCEACAM1 IgV. Larger chemical exchanging effect that is insufficiently suppressed by CPMG method mostly occurs for residues at the GFCC' face.

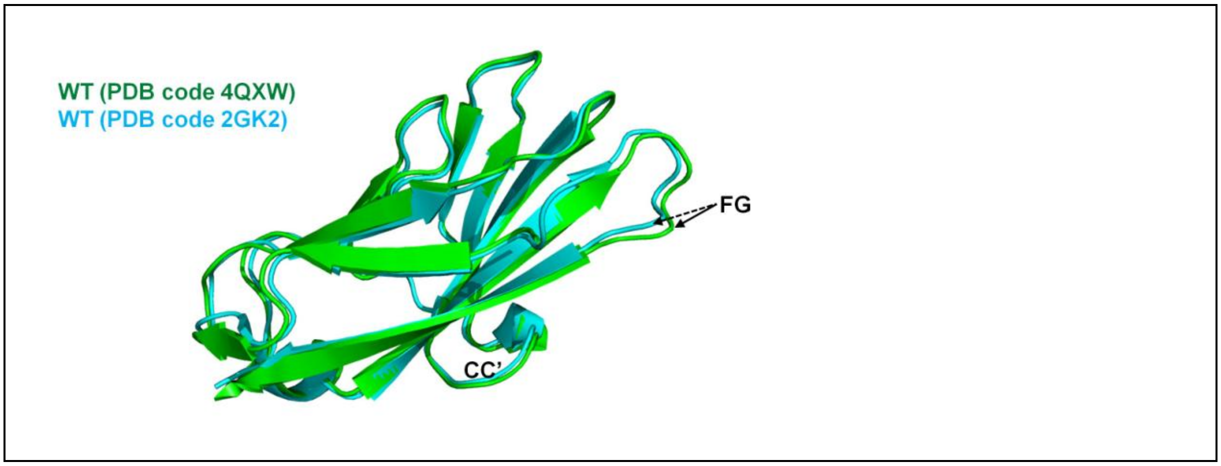

Supplementary Fig. 13 The conformational flexibility of the hCEACAM1 IgV WT GFCC' face. Crystal structures of the hCEACAM1 IgV domain from a GFCC-mediated homodimer crystal (green, PDB code 4QXW) and ABED-mediated homodimer crystal (cyan, PDB code 2GK2) are shown aligned by ribbon representation. CC' and FG loops are labeled, where conformational differences of FG loops in the two crystal structures are indicated by solid and dashed lines.

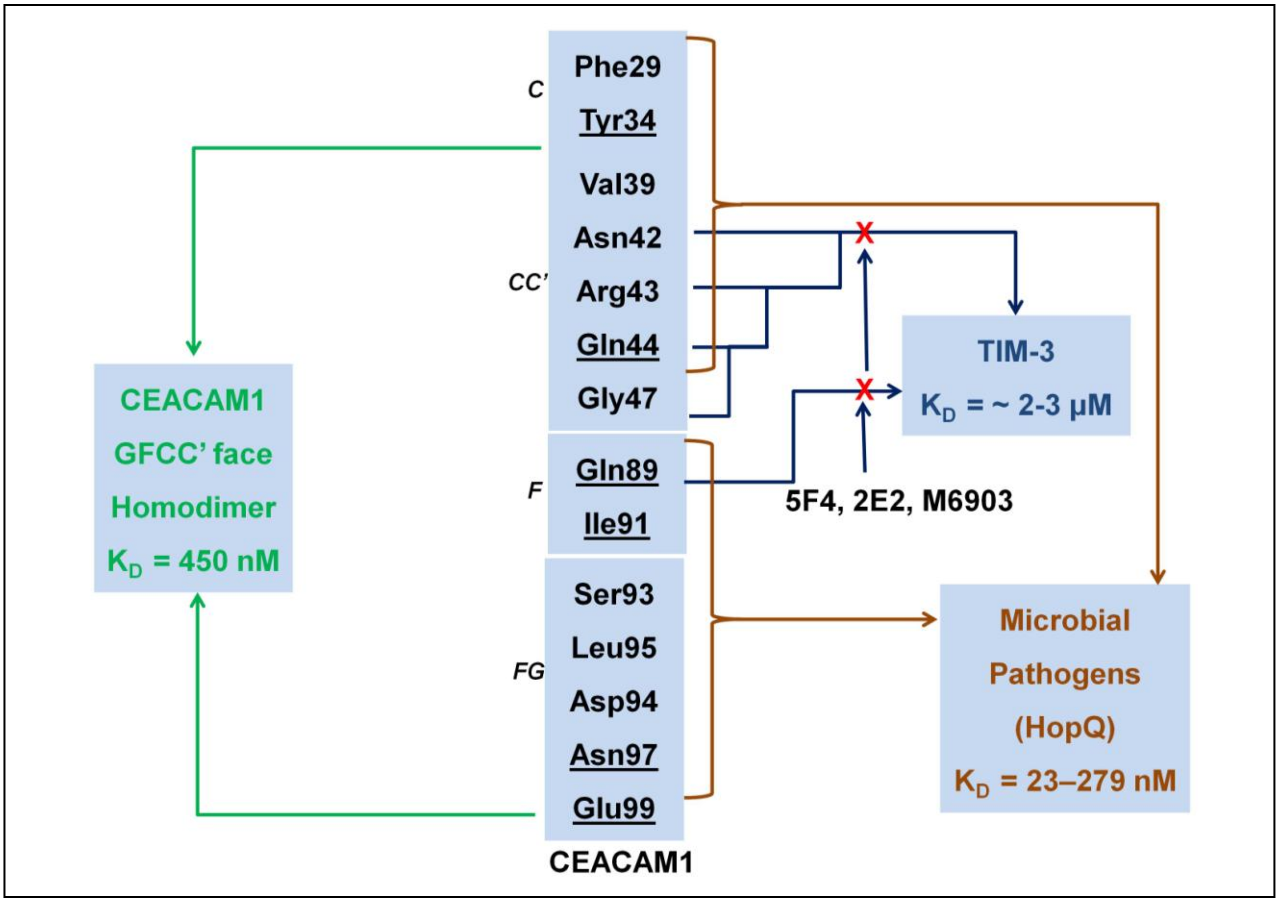

Supplementary Fig. 14. The network of hCEACAM1 residues responsible for mediating homodimer formation and binding with various ligands. The GFCC' face of hCEACAM1 that includes the C strand, CC' loop, F strand and FG loop mediates homodimer formation and interactions with various ligands including hTIM-3 and microbial pathogens such as HopQ. The GFCC' face residues F29, Y34, G41, Q44, Q89, I91, L95, N97 and E99 make hydrophobic and hydrogen bonded interactions with 450 nM affinity (K_D) of homodimer formation[1-2.]. The GFCC' face residues also participate in interactions with hTIM-3 to regulate immune tolerance and exhaustion in which residues N42, R43, Q44, G47 and Q89 mediate the binding with hCEACAM1 as shown by site-directed mutagenesis studies[1]. The binding between hCEACAM1 and hTIM-3 as shown by NMR, SPR and ELISA exhibits a K_D of ~2-3 μM with blockade of this interaction by 5F4 (anti-hCEACAM1 monoclonal), 2E2 (anti-hTIM3 monoclonal antibody) and M6903 (anti-hTIM3 monoclonal antibody), and anti-TIM3 polyclonal

antibodies[2-5.] Further, microbial pathogens such as HopQ[2], also target this GFCC' face for cellular invasion and immune evasion. Specially, the HopQ GFCC' face residues F29, V39, Q44, Q89, I91 and V96 binds hCEACAM1 with a K_D of 23-279 nM that is higher than the K_D associated with homodimer formation and abrogates the GFCC' face associated homodimer formation[2].

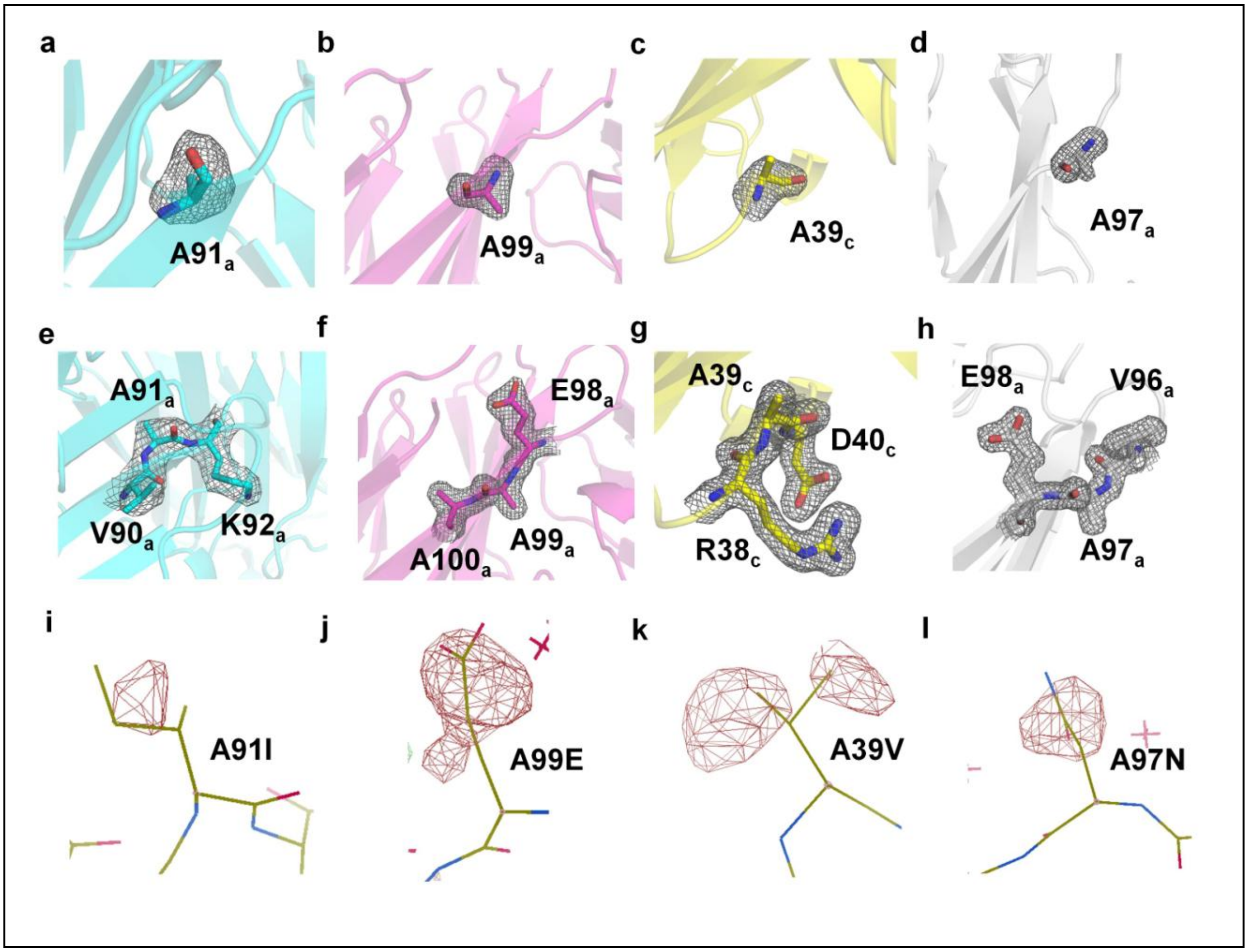

Supplementary Fig. 15 Electron density maps of hCEACAM1 IgV I91A, E99A, V39 and N97A mutants. a-d Fo-Fc map contoured at 3.0 σ level of hCEACAM1 mutants. The electron density map is derived from an initial model where residues A91 (panel a), A99 (panel b), A39 (panel c), and A97 (panel d) were not fitted and significant positive Fo-Fc electron density at 3.0 σ level was observed. Figures a-d showing superimposition of the Fo-Fc map on the final refined model and residues A91 from molecule a (panel a), A99 from molecule a (panel b), A39 from molecule c (panel c), and A97 from molecule a (panel d) are shown by stick representations. The molecule name for the identification of these residues is shown in the subscript. **e-h** 2Fo-Fc map at 1.0 σ level of hCEACAM1 mutants. The electron densiy map is derived from final refined model where residues V90-A91-K92 from molecule a (panel e), E98-A99-A100

from molecule a (panel f), R38-A39-D40 from molecule c (panel g), and V96-A97-E98 from molecule a (panel h) are fitted to the initial model after many rounds of model building and refinement. Figures e-h showing superimposition of 2Fo-Fc map on the final refined model and residues are shown by stick representations. **i-l** Further validation of hCEACAM1 IgV I91A, E99A, V39 and N97A mutants. The final refined model of each mutant was reverse mutated to the respective residue present in the WT, whereas A91I (panel i), A99E (panel j), A39V (panel k), and A97N (panel l) mutations were done in the I91A, E99A, V39 and N97A mutants coordinates, respectively and one round of refinement cycle was performed. The observed negative Fo-Fc map at 3.0 σ level for each mutant clearly shows negative density around reverse mutation site and validates proper model building and refinement of the hCEACAM1 mutants.

Supplementary Table 1: The percent identify matrix of IgV domain of the Human CEACAM family members including CEACAM1 (C1), CEACAM3 (C3), CEACAM4 (C4), CEACAM5 (C5), CEACAM6 (C6), CEACAM7 (C7) and CEACAM8 (C8).

hCEACAM family member (Uniprot ID)	C1	C3	C4	C5	C6	C7	C8
C1|P13688|	100.00	87.96	47.12	88.89	89.81	65.09	72.22
C3|P40198|	87.96	100.00	50.96	86.11	89.81	63.21	68.52
C4|O75871|	47.12	50.96	100.00	47.12	50.96	44.23	50.00
C5|P06731|	88.89	86.11	47.12	100.00	88.89	66.04	71.30
C6|P40199|	89.81	89.81	50.96	88.89	100.00	66.04	72.22
C7|Q14002|	65.09	63.21	44.23	66.04	66.04	100.00	64.15
C8|P31997|	72.22	68.52	50.00	71.30	72.22	64.15	100.00

Supplementary Table 2: The sequence alignments of IgV domain of the Human CEACAM family members including CEACAM1 (C1), CEACAM3 (C3), CEACAM4 (C4), CEACAM5 (C5), CEACAM6 (C6), CEACAM7 (C7) and CEACAM8 (C8).

```
        A     A'         B           C         α1   C'         C"         D        E      α2      F            G
             10         20          30         40         50          60         70         80         90         100
C1  QLTTESMPFNVAEGKEVLLLVHNLPQQLFGYSWYKGERVDGNRQIVGYAIGT-QQATPGPANSGRETIYPNASLLIQNVTQNDTGFYTLQVIKSDLVNEEATGQFHVY
C3  KLTIESMPLSVAEGKEVLLLVHNLPQHLFGYSWYKGERVDGNSLIVGYVIGT-QQATPGAAYSGRETIYTNASLLIQNVTQNDIGFYTLQVIKSDLVNEEATGQFHVY
C4  QFTIEALPSSAAEGKDVLLLACNISETIQAYYWHKGKTAEGSPLIAGYITDI-QANIPGAAYSGRETVYPNGSLLFQNITLEDAGSYTLRTINASYDSDQATGQLHVH
C5  KLTIESTPFNVAEGKEVLLLVHNLPQHLFGYSWYKGERVDGNRQIIGYVIGT-QQATPGPAYSGREIIYPNASLLIQNIIQNDAGFYTLHVIKSDLVNEEATGQFRVY
C6  KLTIESTPFNVAEGKEVLLLAHNLPQNRIGYSWYKGERVDGNSLIVGYVIGT-QQATPGPAYSGRETIYPNASLLIQNVTQNDTGFYTLQVIKSDLVNEEATGQFHVY
C7  QTNIDVVPFNVAEGKEVLLVVHNESQNLYGYNWYKGERVHANYRIIGYVKNISQENAPGPAHNGRETIYPNGTLLIQNVTHNDAGIYTLHVIKENLVNEEVTRQFYVF
C8  QLTIEAVPSNAAEGKEVLLLVHNLPQDPRGYNWYKGETVDANRRIIGYVISN-QQITPGPAYSNRETIYPNASLLMRNVTRNDTGSYTLQVIKLNLMSEEVTGQFSVH
    : . :  * ..****:***:. *  :   .* *:**: ....  * **  .  *   ** * ..** :* *.:**::*:  :* * ***:.*: .  .::.* *: *.
```

* The two α helices and nine β strands are labeled and underlined. The residues are numbered with conserved residues shown in red and non-conserved residues shown in black. Asterisk (*) indicates similar residue conserved across all family members, Period (.) indicates conservation of weakly similar residues, and a colon (:) indicates conservation of strongly similar residues.

Supplementary Table 3: The residues similarity of IgV domain of the Human CEACAM family members including CEACAM1 (C1), CEACAM3 (C3), CEACAM4 (C4), CEACAM5 (C5), CEACAM6 (C6), CEACAM7 (C7) and CEACAM8 (C8).

C1	C3	C4	C5	C6	C7	C8
F29	+	Q	+	I	Y	R
S32	+	Y	+	+	N	N
Y34	+	H	+	+	+	+
V39	+	A	+	+	+	+
D40	+	E	+	+	H	+
G41	+	+	+	+	A	A
N42	+	S	+	+	+	+
Q44	L	L	+	L	R	R
T56	+	I	+	+	A	+
Q89	+	R	H	+	H	+`
I91	+	+	+	+	+	+
N97	+	S	+	+	+	S
E99	+	Q	+	+	+	+

Supplementary Table 4: Quantification metrics of human CEACAM1 WT dimer (PDB code 4QXW) and V39A (PDB code 6XNW), I91A (PDB code 6XNT), N97A (PDB code 6XO1), E99A (PDB code 6XNO), and WT (PDB code 2GK2) crystal structures by PDB-PISA.

Crystal structure	Number of hydrogen bonds	Interface Area (Å^2)	CSS Score
CEACAM1 WT GFCC' face dimer (molecules a/*b,* PDB 4QXW)	17	824.6	0.9
V39A (molecules a/*b*)	16	817.5	1.0
V39A (molecules b/*c*)	4	479.3	0.1
V39A (molecules c/*d*)	5	525.3	0.0
I91(molecules a/*b*)	12	826.0	1.0
N97A (molcules a/*b*)	4	544.5	0.0
E99A (molcules a/*b*)	12	748.6	0.63
CEACAM1 WT (molecules a/*b,* PDB code 2GK2)	3	475.4	0.31

Interface area in Å2, calculated as difference in total accessible surface areas of isolated and interfacing structures divided by two. CSS stands for the Complexation Significance Score, which indicates how significant for assembly formation the interface is.

Supplementary Table 5: Hydrogen bonded interactions as observed in human CEACAM1 WT dimer (PDB code 4QXW) crystal structure.

Interactions	Molecule (a) residues (Interacting atom)	Molecule (*b*) residues (Interacting atom)	Distance (Å)
1	L95[O]	S32[OG]	3.0
2	E99[OE2]	G41[N]	2.9
3	L95[O]	Q44[NE2]	2.8
4	Y34[OH]	Q89[NE2]	3.3
5	Q89[OE1]	Q89[NE2]	3.0
6	Q44[OE1]	N97[N]	3.0
7	S32[OG]	N97[ND2]	2.9
8	Y34[OH]	N97[ND2]	3.0
9	Q89[OE1]	N97[ND2]	3.8
10	S32[OG]	L95[O]	2.9
11	G41[N]	E99[OE1]	2.7
12	Q44[NE2]	L95[O]	2.8
13	Q89[NE2]	Q89[OE1]	3.0
14	Q89[NE2]	Y34[OH]	3.6
15	N97[N]	Q44[OE1]	3.2
16	N97[ND2]	S32[OG]	3.0
17	N97[ND2]	Y34[OH]	3.5

Supplementary Table 6: Hydrogen bonded interactions as observed in E99A (PDB code 6XNO) mutant crystal structure, molecules a/*b.*

Interactions	Molecule (a) residues (Interacting atom)	Molecule (*b)* residues (Interacting atom)	Distance (Å)
1	S32[OG]	N97[ND2]	3.0
2	Y34[OH]	N97[ND2]	3.3
3	Y34[OH]	Q89[NE2]	3.6
4	Q44[OE1]	N97[N]	3.1
5	Q89[OE1]	Q89[NE2]	3.2
6	L95[O]	S32[OG]	2.9
7	L95[O]	Q44[NE2]	2.8
8	S32[OG]	L95[O]	3.0
9	Q 44[NE2]	L95[O]	2.8
10	Q89[NE2]	Q89[OE1]	3.0
11	N97[N]	Q44[OE1]	3.4
12	N97[ND2]	S32[OG]	3.3

Supplementary Table 7: Hydrogen bonded interactions as observed in I91A (PDB code 6XNT) mutant crystal structure, molecules a/*b.*

Interactions	Molecule (a) residues (Interacting atom)	Molecule (*b*) residues (Interacting atom)	Distance (Å)
1	L95[O]	S32[OG]	3.1
2	E99[OE1]	G41[N]	3.2
3	L95[O]	Q44[NE2]	2.8
4	Q89[OQE1]	Q89[NE2]	2.9
5	Q89[OE1]	N97[ND2]	3.4
6	S32[OG]	L95[O]	2.6
7	G41[N]	E99[OE1]	2.9
8	Q44[NE2]	L95[O]	3.0
9	Q89[NE2]	Q89[OE1]	2.8
10	N 97[N]	Q44[OE1]	3.8
11	N97[ND2]	S32[OG]	3.9
12	N97[ND2]	Q89[OE1]	3.6

Supplementary Table 8: Hydrogen bonded interactions as observed in V39A (PDB code 6XNW) crystal structure, molecules a/*b.*

Interactions	Molecule (a) residues (Interacting atom)	Molecule (*b*) residues (Interacting atom)	Distance (Å)
1	L95[O]	S32[OG]	2.9
2	E37[O]	R38[NH1]	3.1
3	E99[OE1]	G41[N]	2.7
4	L95[O]	Q44[NE2]	3.0
5	Q89[OE1]	Q89[NE2]	3.0
6	Q44[OE1]	N97[N]	3.0
7	S32[OG]	N97[ND2]	3.5
8	S32[OG]	L95[O]	3.1
9	R38[NH1]	E37[O]	3.9
10	G41[N]	E99[OE1]	2.7
11	Q44[NE2]	L95[O]	2.8
12	Q89[NE2]	Y34[OH]	3.3
13	Q89[NE2]	Q89[OE1]	3.1
14	N97[N]	Q44[OE1]	3.0
15	N97[ND2]	S32[OG]	3.3
16	N97[ND2]	Y34[OH]	3.2

Supplementary Table 9: Hydrogen bonded interactions as observed in V39A (PDB code 6XNW) mutant crystal structure, molecules b/c.

Interactions	Molecule (*b*) residues (Interacting atom)	Molecule (*c*) residues (Interacting atom)	Distance (Å)
1	E5[OE2]	E16[N]	3.5
2	S72[OG]	Y68[OH]	2.9
3	N70[OD1]	Y68[OH]	3.7
4	N70[ND2]	Y68[OH]	3.8

Supplementary Table 10: Hydrogen bonded interactions as observed in V39A (PDB code 6XNW) mutant crystal structure, molecules c/*d.*

Interactions	Molecule (c) residues (Interacting atom)	Molecule (*d)* residues (Interacting atom)	Distance (Å)
1	D 94[O]	Q44[NE2]	3.1
2	N97[OD1]	Q89[NE2]	3.3
3	Q44[NE2]	D94[O]	2.9
4	N97[ND2]	Q89[OE1]	2.8
5	N97[ND2]	N97[OD1]	2.4

Supplementary Table 11: Hydrogen bonded interactions as observed in N97A (PDB code 6XO1) mutant crystal structure, molecules a/*b.*

Interactions	Molecule (a) residues (Interacting atom)	Molecule (*b)* residues (Interacting atom)	Distance (Å)
1	N26[OE1]	I67[N]	2.9
2	N26[NE2]	I67[O]	2.9
3	N53[NE2]	G58[O]	3.9
4	N53[NE2]	N61[O]	3.5

Supplementary Table 12: Hydrogen bonded interactions as observed in human CEACAM1 WT (PDB code 2GK2) crystal structure, molecules a/*b.*

Interactions	Molecule (b) residues (Interacting atom)	Molecule (a) residues (Interacting atom)	Distance (Å)
1	E16[OE1]	N70[ND2]	2.2
2	S72[OG]	Y68[OH]	2.6
3	N70[ND2]	Y68[OH]	2.6

Supplementary References

1. Huang, Y. H. et al. CEACAM1 regulates TIM-3-mediated tolerance and exhaustion. Nature 517, 386–390 (2015).
2. Bonsor, D. A. *et al.* The Helicobacter pylori adhesin protein HopQ exploits the dimer interface of human CEACAMs to facilitate translocation of the oncoprotein CagA . *EMBO J.* (2018). doi:10.15252/embj.201798664
3. Huang, Y.-H. *et al.* Erratum: Corrigendum: CEACAM1 regulates TIM-3-mediated tolerance and exhaustion. *Nature* (2016). doi:10.1038/nature17421
4. Gandhi, A. K. *et al.* High resolution X-ray and NMR structural study of human T-cell immunoglobulin and mucin domain containing protein-3. *Sci. Rep.* (2018). doi:10.1038/s41598-018-35754-0
5. Zhang, D. *et al.* Identification and characterization of M6903, an antagonistic anti–TIM-3 monoclonal antibody. *Oncoimmunology* **9**, (2020).
6. Sabatos-Peyton, C. A. *et al.* Blockade of Tim-3 binding to phosphatidylserine and CEACAM1 is a shared feature of anti-Tim-3 antibodies that have functional efficacy. *Oncoimmunology* (2018). doi:10.1080/2162402X.2017.1385690
