# Supplementary material for: Structural basis of the dynamic human CEACAM1 monomer-dimer equilibrium
Source: Commun Biol. 2021 Mar 19;4:360. doi: 10.1038/s42003-021-01871-2 (PMC7979749; doi:10.1038/s42003-021-01871-2)
Supplement: Supplementary file 3 — Description of Additional Supplementary Files [file 42003_2021_1871_MOESM3_ESM.pdf]

Description of Additional Supplementary Files

File name: Supplementary Data 1

Description: Source data underlying plots shown in Fig. 1, Fig.6, Fig. 7, Supplementary Fig. 3, and Supplementary Fig. 12.
